# Strategies to Refine Gastric Stimulation and Pacing Protocols: Experimental and Modeling Approaches

**DOI:** 10.3389/fnins.2021.645472

**Published:** 2021-04-22

**Authors:** Leo K. Cheng, Nipuni D. Nagahawatte, Recep Avci, Peng Du, Zhongming Liu, Niranchan Paskaranandavadivel

**Affiliations:** ^1^Auckland Bioengineering Institute, University of Auckland, Auckland, New Zealand; ^2^Department of General Surgery, Vanderbilt University Medical Center, Nashville, TN, United States; ^3^Riddet Institute, Palmerston North, New Zealand; ^4^Department of Biomedical Engineering, University of Michigan, Ann Arbor, MI, United States; ^5^Department of Electrical Engineering and Computer Science, University of Michigan, Ann Arbor, MI, United States

**Keywords:** stimulation, pacing, gastric motility, slow wave, biophysical model, mathematical model

## Abstract

Gastric pacing and stimulation strategies were first proposed in the 1960s to treat motility disorders. However, there has been relatively limited clinical translation of these techniques. Experimental investigations have been critical in advancing our understanding of the control mechanisms that innervate gut function. In this review, we will discuss the use of pacing to modulate the rhythmic slow wave conduction patterns generated by interstitial cells of Cajal in the gastric musculature. In addition, the use of gastric high-frequency stimulation methods that target nerves in the stomach to either inhibit or enhance stomach function will be discussed. Pacing and stimulation protocols to modulate gastric activity, effective parameters and limitations in the existing studies are summarized. Mathematical models are useful to understand complex and dynamic systems. A review of existing mathematical models and techniques that aim to help refine pacing and stimulation protocols are provided. Finally, some future directions and challenges that should be investigated are discussed.

## Introduction

Bioelectricity is a key regulator of bodily function. The coordinated contraction and relaxation of the muscles necessary for controlling movement are initiated by electrical signals propagating through the nerves and muscles. Disorders in the electrical signals and patterns can result in dysmotility. An improved understanding of how intrinsic bioelectrical events, and externally introduced electrical signals, interact with our nerves and muscles is critical for refining existing, and developing new therapies. The foundations of electrophysiology can be attributed to Galvani’s studies on frog muscles and hearts in the 1790s (Piccolino, 1997). It was not long afterward that the first reports of using electrical stimulation to treat cardiac conditions occurred in the 1800s, despite the underlying science being uncertain (Green, 1872; McWilliam, 1889). Subsequently, in the 1920s, the first external cardiac pacemakers were reported independently by Lidwell and Hyman (Aquilina, 2006; Mond et al., 2012). Hyman was the first to coin the term “artificial pacemaker,” a term that is still widely used to date.

Bioelectrical signals were first measured from the gastrointestinal tract in the 1920s (Alvarez, 1922b). In his seminal studies, Alvarez established that rhythmic electrical events occurred 3 cycles per minutes (cpm) in the human stomach. These electrical events were omnipresent, and occurred even in the absence of any visible contractions (Alvarez, 1922a). Despite substantial effort devoted to investigate the normal and impaired bioelectrical activity along the gastrointestinal tract over the last century, our understanding of gastrointestinal electrophysiology and the application of electrical pulses to treat disorders has lagged the cardiac field (Cheng, 2015; Avci et al., 2020). This is partly because the gastrointestinal tract has a number of cooperating and overlapping mechanisms that coordinate the mechanical contractions to aid in the breakdown, mixing and transportation of luminal contents (Huizinga and Lammers, 2009). One of the key contributors is bioelectrical events, known as slow waves, that are generated by interstitial cells of Cajal (ICC) (Huizinga et al., 1995). Loss of ICC and disordered slow wave patterns have been associated with a number of functional gastrointestinal and motility disorders (see section “Gastric Functional Motility Disorders”). Therefore, the use of external electrical impulses to restore function is an attractive approach.

Electrical stimulation of the stomach was first reported in 1963 as a potential therapy for paralytic ileus–the loss of peristaltic activity that frequently occurs following abdominal surgery (Bilgutay et al., 1963). Investigators have also applied electrical impulses to treat gastroparesis by improving the rate of gastric emptying (McCallum et al., 1998), or to treat obesity, by delaying the rate of gastric emptying (Sarna et al., 1976; Liu et al., 2006a). However, the results have generally been inconsistent and variable. This variability can be associated with a number of key problems: (i) lack of understanding of the mechanisms of action, (ii) large variation in the pulses sequences applied, (iii) lack of high-quality human trials, and (iv) lack of unbiased methods for quantifying the efficacy of a therapy.

In recent years, there has been renewed interest in the use of electrical impulses to modulate the bioelectrical activity for acute and chronic disorders. Neurology and cardiology have received the most attention, but it has also been applied for pain management and for the treatment of functional gastrointestinal motility disorders. This review will focus on the application of electrical impulses directly on the stomach musculature and techniques used to optimize the pacing parameters. In addition, some future directions for stimulation, pacing and modeling efforts are discussed.

## Gastric Functional Motility Disorders

Gastric motility disorders are complex and often present with overlapping symptoms and phenotypes of disorders. Some functional motility disorders that are associated with disordered slow wave activity, along with obesity and post-operative ileus are discussed below.

### Gastroparesis

Gastroparesis is a chronic symptomatic gastric motility disorder, defined as delayed gastric emptying of a solid meal in the absence of mechanical obstruction. Symptoms or signs of gastroparesis include nausea, vomiting, early satiety, fullness, anorexia, and/or weight loss (Camilleri et al., 2013). Definitive diagnosis for gastroparesis includes retention of greater than 10% of a solid-meal as determined by scintigraphy after 4 h (Abell et al., 2008). Gastroparesis is not a common disease, with its prevalence reported at 0.2% in the United States (Syed et al., 2020). However, delayed emptying has been estimated to be 1.8% in the community and suggests that gastroparesis may be undiagnosed in many subjects in the population. Patients with severe gastroparesis may require long-term enteral or parenteral nutritional support, as well as frequent and prolonged hospital admissions (Wang et al., 2008).

Gastroparesis is associated with loss and degradation of the ICC network (Farrugia, 2008; Grover et al., 2011). The NIH Gastroparesis Clinical Research Consortium^1^ has collected and analyzed full-thickness gastric biopsies from gastroparetic patients undergoing gastric stimulator implantation and reported a 48% reduction in ICC numbers in diabetic gastroparesis patients compared to age matched controls (2.8 vs. 5.3 cells/high powered field) (Grover et al., 2011). The degradation of ICC numbers and network integrity are associated with disordered slow wave and motility patterns (see section “High Resolution Slow Wave Mapping”). Therefore, a variety of electrical therapies have been proposed to treat gastroparesis (Paskaranandavadivel et al., 2020). Therapies have reported to both improve symptoms (McCallum et al., 2010, 2013; Ducrotte et al., 2020), and in some cases improve gastric emptying rates (McCallum et al., 1998) (see section “Gastric Stimulation and Pacing” for further details).

### Chronic Unexplained Nausea and Vomiting

Chronic unexplained nausea and vomiting patients have symptoms similar to gastroparesis patients (Pasricha et al., 2011). However, they have normal gastric emptying (less than 10% retention of a solid meal after a 4 h period). These patients have been reported to have a moderate reduction in ICC numbers compared with controls, with gastroparesis patients having the greatest reduction in ICC numbers (5.6 vs. 3.5 vs. 2.3 cell bodies/high powered field) (Angeli et al., 2015). In addition, compared with gastroparesis, patients with chronic unexplained nausea and vomiting have less smooth muscle fibrosis (Bashashati et al., 2017). Disordered slow wave patterns have also been reported in these patients (see section “High Resolution Slow Wave Mapping”).

### Functional Dyspepsia

Functional dyspepsia is defined by the Rome IV guidelines when a subject has presented with post-prandial fullness, early satiety, epigastric pain and epigastric burning (Futagami et al., 2018). It is also associated with impaired accommodation of the proximal stomach, early distension and impaired motility of the antrum post-prandial (Bortolotti et al., 1995; Gilja et al., 1996). The reported prevalence of functional dyspepsia is variable from 5 to 40%, with varying severities in symptoms (Enck et al., 2017).

Cutaneous electrogastrography studies have detected slow wave frequencies outside the normal range (i.e., greater or less than 2–4 cpm) in functional dyspepsia subjects. In one study, abnormal frequencies were detected in 83% of the subjects (Sha et al., 2009). A recent high resolution (HR) electrogastrography study was able to detect spatial slow wave abnormalities in 44% of subjects along with a higher proportion of aberrant propagation directions (Gharibans et al., 2019). In addition, abnormal spatial parameters were found to be correlated with severity of upper gastrointestinal symptoms. Electrical therapies using implanted and transcutaneous methods have successfully been applied to relieve symptoms (Lu et al., 2013; Zhu et al., 2021).

### Obesity

Obesity is a significant risk factor for a range of diseases from diabetes, cardiovascular disease to cancer (Sowers, 2003). It is also associated with a host of gastrointestinal symptoms from abdominal pain, bloating, diarrhea, and frequency vomiting (Delgado-Aros et al., 2004). Its prevalence has significantly increased, with reports of 20–40% of the population being obese across various countries (Hales et al., 2020). Typical management involves physical activity, modifying eating patterns, reducing caloric intake, drug therapy and counseling (Turner et al., 2018). In severe cases, bariatric surgery is considered, where the stomach capacity is reduced to limit food intake or bypassed to reduce nutrient uptake. Electrical therapies have been proposed as a treatment for obesity. However, mixed results have been reported, with some human trials reporting promising results (Cigaina, 2004; Favretti et al., 2004; Yao et al., 2005; Liu et al., 2006a), but longer term studies have failed to show sustained weigh loss over an extended period (Johannessen et al., 2017).

### Post-operative Ileus

Post-operative ileus, a transient impairment of gastrointestinal motility, is a common occurrence after abdominal surgery and has limited treatment options (Iyer et al., 2009). It is associated with delayed recovery, prolonged hospital stays, significant increases in health costs and increased risks of morbidity (Iyer et al., 2009). Patients may experience abdominal pain, nausea, vomiting, bloating, and are unable to eat or pass stool for multiple days (Vather et al., 2013). Due to these needs, post-operative ileus was proposed as one of the initial targets for electrical therapy (Bilgutay et al., 1963; Hocking et al., 1992).

## Electrical Conduction System of the Stomach

The cause, diagnosis and treatments of GI functional motility disorders as described in section “Gastric Functional Motility Disorders” remain poorly understood. A number of these disorders are linked with impaired electrophysiology of the GI tract. Therefore, an improved understanding of the electrical conduction system in the GI tract, in both health and disease, will assist in the development of new and the refinement of existing therapies.

Slow waves are a key input that are generated and propagated by ICC (Huizinga et al., 1995). ICC are named after Nobel laureate Santiago Ramón y Cajal. In the late 19th century, Cajal described a new type of nerve-like branching networks interleaved among neurons embedded within the smooth muscle linings in the GI tract (Cajal, 1911). However, their developmental origin and function remained unclear for many decades (Huizinga et al., 1995). Subsequently, Thuneberg sparked a revival in ICC research by proposing that ICC were the intestinal pacemaker cells (Thuneberg, 1982) and showed that injury of ICC resulted in loss of slow wave activity (Liu et al., 1994).

Individually, ICC have different intrinsic frequencies, however, *in vivo* they form an integrated network and entrain to the dominant highest frequency (Hinder and Kelly, 1977; Hirst and Edwards, 2006). For example, stomach ICC have been found to have a gradient of intrinsic activity (Xue et al., 1995; Hirst and Edwards, 2006). However, in the normal, intact human stomach, all activity is entrained to a dominant frequency of 3 cpm (Hinder and Kelly, 1977; O’Grady et al., 2010a). If distal regions are either partially or totally isolated, they autonomously generate slow waves at a lower intrinsic frequency (Sarna et al., 1972; Hinder and Kelly, 1977). In addition, if tissue samples are excised and left for an extended period of time, the slow wave frequency can increase significantly higher than the normal *in vivo* frequency. For example, in the cat stomach the frequency increased from 4.1 to 12.0 cpm (Xue et al., 1995) and in excised human tissue samples, frequency was reported to be as high as 7.4 cpm in the antrum, while the excised fundus (normally devoid of cyclic activity) had a frequency of 5.1 cpm (Rhee et al., 2011). Therefore, *in vivo* slow wave recordings are likely to be a more accurate representation of intrinsic physiology (Angeli et al., 2013; Sarna, 2013; Huizinga, 2017).

Slow waves depolarize the neighboring smooth muscle cells, either through gap junctions or peg-and-socket coupling (Torihashi et al., 2002; Huizinga et al., 2010). Under the right combination of neuro-hormonal conditions, the depolarized smooth muscle cells are able to generate contractions based on a series of intracellular Ca^2+^ release mechanisms (Karaki and Weiss, 1984). The sustained plateau of depolarization of smooth muscle membrane can also lead to bursts of action potentials or spikes that are associated with Ca^2+^ entry and contractile activity (Ver Donck et al., 2006; Lee et al., 2007).

Importantly, the stomach is not completely autonomous (Travagli and Anselmi, 2016). Slow wave activity is coordinated by the ICC, but the resulting motility is also modulated through extrinsic nerve innervation (Powley et al., 2019), and the enteric nervous system (Furness, 2012; Furness et al., 2014). In this regard, the vagus plays a central role in extrinsic neural control of the stomach. The nucleus tractus solitarius is the primary viscerosensory nucleus of the vagus, which gathers vagal afferent input and modulates the dorsal motor nucleus of the vagus to alter visceromotor output. The left vagal fibers are understood to innervate the proximal region of the stomach where the gastric pacemaker is located (Pagani et al., 1988). Additionally, the vagus nerve can modulate antral motility, pyloric opening, and gastric emptying (Lu et al., 2018, 2020). Neuropathy has also been known to accompany a number of gastrointestinal diseases, particularly when accompanied by diabetes (Vinik et al., 2003). Animal models of impaired gastrointestinal functions have also shown damage to both extrinsic and intrinsic nerves, as well as reduced ICC (Young and Edwards, 2005). The readers are referred to Furness et al. (2014) for a detailed review of the vagal control of gut motility, and Phillips et al. (2006) for a detailed review of neuropathy affecting the gastrointestinal tract.

## Slow Wave Activity in the Stomach

### Slow Wave Recordings

**FIGURE 1:**
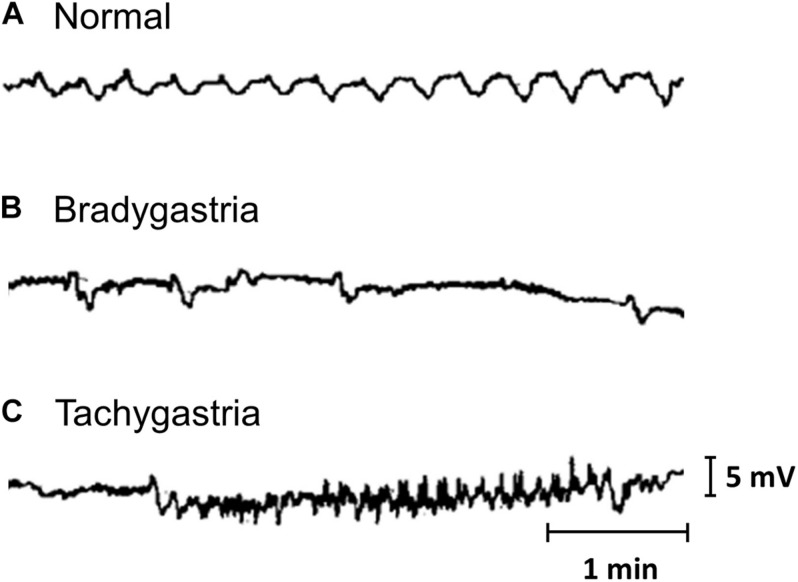
Gastric mucosal slow wave traces of **(A)** normal, **(B)** bradygastric, and **(C)** tachygastric activity. Modified from Abell and Malagelada (1985).

The earliest recordings of gastrointestinal slow wave activity can be attributed to the foundational work of Alvarez (1922b, c). Initially, measurements were obtained from individual locations, either on the surface of the stomach or on the body surface. Such recordings allowed the normal frequency of the slow wave events to be established. The term tachygastria was first introduced in 1974, referring to activity with an abnormally high frequency (Code and Marlett, 1974). Bradygastria, a term for low frequency slow wave activity, was soon reported in human studies (You and Chey, 1984; Abell and Malagelada, 1985). Examples of normal, bradygastric and tachygastric slow wave traces are shown in Figure 1. The frequency bands of gastric slow wave activity types have now been formally defined, with normal activity generally defined to be within 2.5–3.75 cpm and bradygastria and tachygastria, below or above this range (Parkman et al., 2003; Koch, 2011).

Subsequently, more electrodes were introduced and by placing the electrodes along the central axis of the stomach, it was possible to track the propagation of the slow wave from electrode to electrode (Kelly et al., 1969; Hinder and Kelly, 1977). Using these techniques, propagation direction and speed were able to be deduced. However, it is now clear that such sparse or low resolution slow wave recordings can at times be misleading due to spatial aliasing, especially when activity is dysrhythmic (Cheng et al., 2013; O’Grady et al., 2019).

### High Resolution Slow Wave Mapping

High resolution mapping utilizes hundreds of spatially dense electrodes, typically arranged in a two-dimensional grid, to map the spatio-temporal pattern of slow wave activity. Adapting techniques that had previously been used to map the electrical activity in the heart (Durrer et al., 1970; Pullan et al., 2003), HR mapping was first applied to the gastrointestinal tract by Lammers et al. (1993), to map the slow wave activity in the rabbit duodenum. Similar methods have now been applied to map the slow wave patterns in the stomach of a variety of animal species (Lammers et al., 1998, 2009; Du et al., 2009a; Egbuji et al., 2010), and the human stomach in both health and disease (O’Grady et al., 2010a, 2012a; Angeli et al., 2015). HR mapping studies have identified regional variations in gastric slow wave activity, most notably a region of high amplitude and high velocity in the distal antrum of the human stomach (Berry et al., 2016). It is postulated that this distal antral wave acceleration, along with coordination with the pylorus, plays a major role in retropulsion of contents to allow both efficient mixing and breakdown on contents, along with antral flow of contents into the intestine (Berry et al., 2016; Ishida et al., 2019).

As discussed in section “Gastric Functional Motility Disorders,” a loss of ICC numbers and a breakdown in the ICC network integrity has been reported in gastroparesis patients (Grover et al., 2011). Furthermore, HR mapping studies in gastroparetic and chronic unexplained nausea and vomiting patients undergoing Enterra stimulator implantation (Medtronic Inc., Minneapolis, MN, United States) reported a range of abnormal slow wave propagation patterns including, ectopic pacemakers and conduction blocks (O’Grady et al., 2012a; Angeli et al., 2015). Mechanism of slow wave dysrhythmias can be classified into disorders of: (i) conduction and (ii) initiation (O’Grady et al., 2014). Ectopic pacemakers are an example of an initiation disorder, where a secondary pacemaker may compete with the primary or normal pacemaker. In this case, the ectopic pacemaker may initiate retrograde waves that eventually collide with waves generated by the natural pacemaker located in the upper/mid-corpus leading to uncoordinated or uncoupled activity in different regions of the stomach (Berry et al., 2017). A conduction block is an example of a conduction abnormality. In this case, a region of tissue may have a zone of abnormally slow propagation, or force the slow wave to propagate around the conduction block zone. This may then result in secondary forms of dysrhythmias, including reentrant activity and rapid circumferential activation (Lammers et al., 2008; O’Grady et al., 2011). Rapid circumferential propagation is present in a range of gastric dysrhythmias, elevating extracellular amplitudes and organizing transverse wavefronts that may help to reset activity back into an ordered band (O’Grady et al., 2012b). One important finding from the HR mapping studies was that abnormalities of slow wave initiation and conduction occurred at a frequency similar to normal slow wave patterns (around 3 cpm). As a result, such spatial abnormalities could be missed using techniques that lack sufficient spatial resolution (Buist et al., 2006).

**FIGURE 2:**
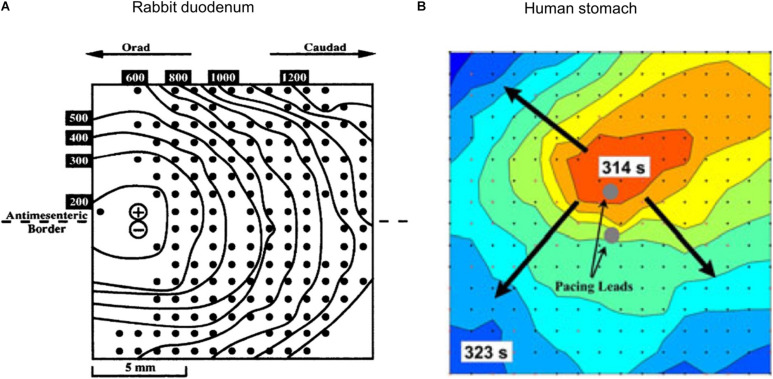
Combining HR mapping and pacing techniques allows the slow wave response to be tracked and quantified. **(A)** Pacing on the left side of a rabbit duodenum preparation and resultant propagation across the mapped area, **(B)** Pacing from the center of a 60 mm^2^ electrode array (red is early activation, and blue is late activation), and corresponding radial propagation from the pacing leads (gray circles). Modified from Lammers et al. (1993) and Alighaleh et al. (2018).

By combining HR mapping with pacing or stimulation techniques, it is possible to monitor and track the electrophysiological response of the organ. The first study to report such techniques was conducted in the isolated rabbit duodenum (see Figure 2A; Lammers et al., 1993). Similar techniques have been applied to pigs to investigate the effects of different pacing parameters (O’Grady et al., 2010b), to test the efficacy of new pacemaker designs (Wang et al., 2018; Alighaleh et al., 2019) and to assess acute slow wave response in patients undergoing Enterra stimulator implantation (Angeli et al., 2016). As Enterra Therapy uses high-frequency stimulation protocols, its mechanism of action is likely via nerves. Despite using a variety of stimulation parameters, including protocols that attempted to mimic pacing parameters within hardware limitations (e.g., amplitude 19 mA, pulse-width 0.45 ms, frequency 130 Hz, on period 1 s, off period 17 s), no changes in slow wave patterns were observed during these acute studies (Angeli et al., 2016). Initial work on mapping the slow wave response with pacing protocols has been applied to human studies (Alighaleh et al., 2018). An example of such results is shown in Figure 2B, where pacing electrodes were integrated into the center of the HR electrode array and a new pacemaker region was initiated slightly above the pacing electrodes (identified by the dark orange area). Further work is required to assess regional electrophysiological characteristics and long-term outcomes.

## Gastric Stimulation and Pacing

**FIGURE 3:**
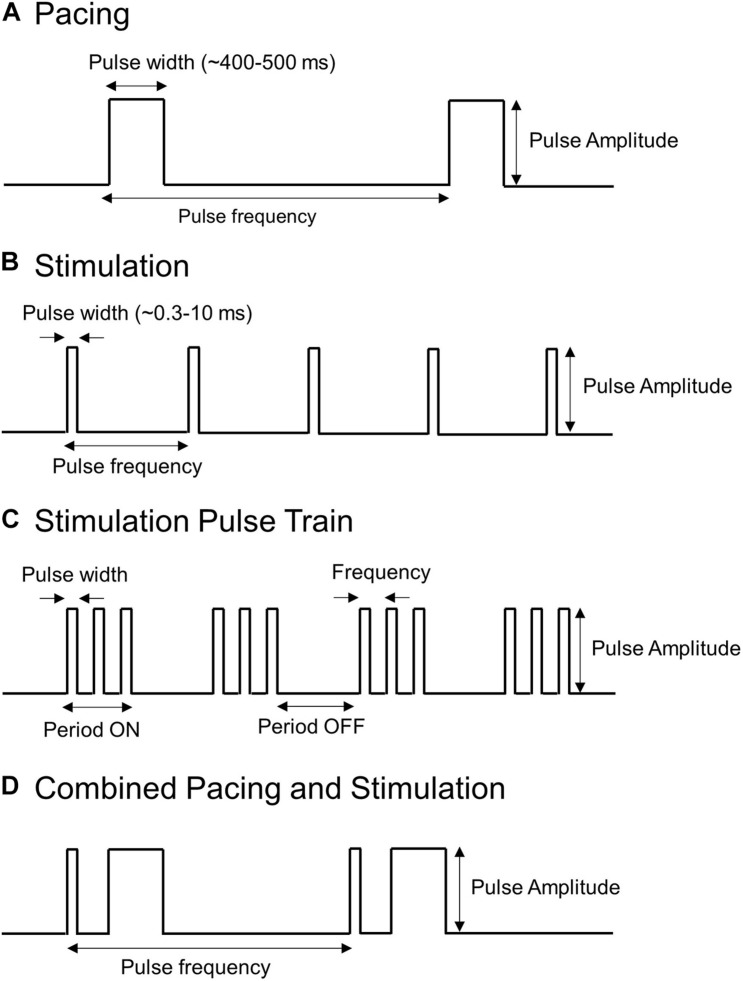
Types of pulses used to stimulate or pace the stomach. Shown are **(A)** Long pulses associated with pacing (high-energy and low frequency), **(B,C)** Short pulses or trains of short pulses associated with stimulation (low energy and high frequency), and **(D)** a hybrid pacing and stimulation approach that combines short and long pulses.

Electrical pulses applied to the stomach can take many forms, which typically consist of a series of rectangular pulses applied as a constant current or voltage at a prescribed frequency. They can be broadly grouped into two categories: (i) pacing methods that are typically low frequency and high energy and (ii) stimulation methods that are typically high frequency and low energy. Figure 3 illustrates some of the types of electrical pulses that have been applied to the stomach. Key parameters that define the electrical pulses include the amplitude, pulse width, frequency at which pulses are applied. In some cases, stimulation pulses are modulated or comprised of trains of shorter pulses.

### Gastric Pacing

Pacing methods involve the application of high energy pulses applied at a frequency close to the intrinsic slow wave frequency. These methods attempt to entrain or modulate the underlying slow wave activity of the stomach, hence they primarily target the ICC to alter slow wave patterns, and therefore motility patterns.

**FIGURE 4:**
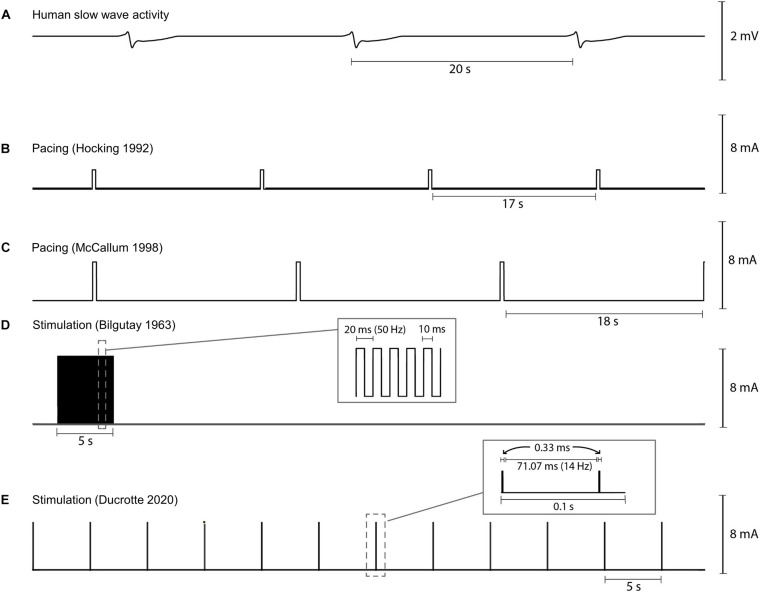
Illustration of variability in pacing and stimulation pulses used by various researchers for human gastric pacing studies. **(A)** Normal gastric slow wave activity occurring at 3 cpm, **(B,C)** gastric pacing parameters as applied by Hocking et al. (1992) (2 mA, 350 ms, IF + 0.5 cpm) and McCallum et al. (1998) (4 mA, 350 ms, 110% IF), and **(D,E)** gastric stimulation parameters as applied by Bilgutay et al. (1963) (7 mA, 50 Hz, 5 s on period, 55 s off period) and Ducrotte et al. (2020) (5 mA, 14 Hz, 0.1 s on period, 5 s off period). IF: intrinsic frequency.

The pulse sequences used for two human gastric pacing protocols (Hocking et al., 1992; McCallum et al., 1998) are visually compared in Figure 4. Pacing methods were initially trialed in dog studies and showed the ability to either increase or decrease the slow wave frequency (intrinsic frequency 5.0 cpm, modulated range 4.2–8 cpm) in response to the pacing (Kelly and La Force, 1972). Subsequently, human studies were reported by two groups in 1992 (Hocking et al., 1992; Miedema et al., 1992). Hocking et al. (1992) were one of the first to apply pacing protocols to the stomach applying a pulse at 2 mA for 300 ms at between 0.3 and 1.6 cpm higher than the intrinsic slow wave frequency. They were able to entrain and increase the slow wave frequency but were unable to improve gastric emptying. However, other studies have reported that gastric emptying could be enhanced with gastric pacing (Eagon and Kelly, 1993; McCallum et al., 1998). McCallum et al. (1998) applied 4 mA for 300 ms at 110% of the intrinsic frequency of baseline slow waves to gastroparesis patients and were able to correct tachygastric slow waves and also improve gastric emptying rates.

On the other hand, Sarna et al. (1976) demonstrated that gastric emptying could be delayed by pacing from the distal antrum in dogs. Therefore, the use of pacing protocols to treat obesity has also been investigated (Cigaina et al., 1996). Such methods aim to limit food intake by reducing rates of gastric emptying. Although exact mechanisms remain uncertain, likely contributors include inhibiting antral contractions or initiating retrograde slow wave to disrupt the intrinsic slow wave activity. Trials in a small human cohort have shown promising results (Cigaina, 2004; Favretti et al., 2004; Yao et al., 2005; Liu et al., 2006a).

Table 1 summarizes the pacing protocols used in both human and animal studies. A typical range of effective pacing parameters reported in the literature across species can be summarized as 2–5 mA of amplitude and 300–500 ms pulse width with a frequency similar to intrinsic frequency. Applying correct pulse width and amplitude is essential for an effective pacing. In humans, 4–5 mA current and 300 ms pulse width applied at a frequency similar to, or larger than, the intrinsic frequency were reported to be the most effective parameters. On the other hand, lower amplitudes (e.g., 0.5–1 mA) and pulse widths (e.g., 3 or 30 ms) were reported to fail to entrain slow wave activity (Lin et al., 1998, 2011). The amplitude and pulse width parameters used in other species such as pig, dog, and rats were relatively comparable with human studies. Since the baseline slow wave activity is to be modulated via pacing, the frequency of applied pulse is also a critical factor for effective pacing and likely vary between subjects and species.

**TABLE 1:** Protocols used for gastric pacing.

References	Species	Amplitude	Pulse Width	(Applied) Frequency
Miedema et al., 1992	Human	4 V	60 ms	IF + 0.5 cpm
Hocking et al., 1992	Human	2 mA	300 ms	IF + (0.3–1.6) cpm
Lin et al., 1998	Human	4 mA	300 ms	110% IF
McCallum et al., 1998	Human	4 mA	300 ms	110% IF
Yao et al., 2005	Human	5 mA	500 ms	9 cpm
Lin et al., 2011	Human	4 mA	350 ms	110% IF
		2 and 0.5 mA*	150 and 150 ms	110% IF
Kelly and La Force, 1972	Dog	1–8 mA	100–2000 ms	2–12 cpm
Sarna and Daniel, 1973	Dog	1–25 V	100 ms	4.8–7.3 cpm
Bellahsene et al., 1992	Dog	2 mA	300 ms	IF (approximately 5 cpm)
Song et al., 2005	Dog	5 mA	550 ms	110% IF
		1 and 0.6 mA*	200 ms	110% IF
Xu et al., 2008	Dog	4 mA	300–500 ms (Modulated at 50 Hz)	110% IF
		1 and 0.5 mA*	200–400 ms (modulated at 50 Hz)	110% IF
O’Grady et al., 2010b	Pig	4 mA	400 ms	Similar to, or faster than IF
Wang et al., 2018	Pig	5 and 8 mA	500, 900 ms	2.7–6 cpm
Alighaleh et al., 2019	Pig	4 mA	400 ms	110% IF
Yin et al., 2006	Mouse	3 mA	20–150 ms	110% IF

IF: intrinsic frequency.

*: two channel pacing.

### Gastric Stimulation

Stimulation methods apply higher frequency pulses at lower energy level compared with pacing techniques. They do not seek to entrain the underlying slow wave patterns but instead target the neural pathways that also have a role in modulation of motility patterns.

Bilgutay et al. (1963) were one of the first to report the concept of gastric electrical stimulation where a pulse sequence with amplitudes of 7–10 mA at 50 Hz for 5 s periods was applied in each minute (see Figure 4D). They reported results from 5 case studies out of 40 patients with paralytic ileus. Using their methods, stimulation was successful in generating peristalsis in post-operative ileus following a variety of abdominal operations. Stimulation resulted in early return of bowel activity, reduced the time for intravenous administration of fluids and electrolytes, allowed oral intake to resume sooner, and reduced the passing of flatulence from 55 to 20 h.

Familoni et al. (1997a) performed one of the early stimulation studies in dogs. They used short pulses of 0.3 ms duration and 2 mA amplitude, and were able to both entrain the slow wave activity and elicit contractions. When the pulses were applied at 20 or 30 cpm (0.33 or 0.5 Hz) the motility patterns were significantly enhanced. Similar techniques were then attempted on a refractory gastroparesis patient (Familoni et al., 1997b). They found that stimulation at a frequency much greater than the intrinsic frequency (i.e., at 12 cpm) resulted in improved emptying and symptoms in that patient. As a result of these promising results, the World-wide Anti-Vomiting Electrical Stimulation study (WAVESS) was performed (Abell et al., 2002, 2003). During the initial double blinded phase of WAVESS, there was a significant reduction in the frequency of vomiting. After 12 months, there was a greater than 50% reduction in vomiting in more than 70% of subjects, and vomiting and nausea severity scores were significantly improved (Abell et al., 2003). As a result, the Enterra Therapy system was developed and approved for use under humanitarian device exception by the FDA.

For detailed meta-analysis of past high-frequency stimulation trials for treating gastroparesis refer to Table 1 in O’Grady et al. (2009) and Table 1 in Chu et al. (2012). As part of these analyses only two randomized, control trials were reported and both were classified as “medium quality” (Abell et al., 2003; McCallum et al., 2010). Recently, Ducrotte et al. (2020) completed a comprehensive randomized control study evaluating the standard Enterra Therapy pulse sequence of 5 mA, 14 Hz pulse train applied for 0.1 s, followed by a 5 s off period (see Figure 4E). It should be noted that the pulse train only consisted of 2 impulses separated by 71 ms because of the short 0.1 s on period. The study determined that the stimulator was effective in reducing the frequency of vomiting and nausea in a subset of patients. However, overall after 4 months of gastric stimulation, neither gastric emptying nor quality of life was improved. Nevertheless, patients with medically resistant symptoms may benefit from gastric stimulation to relieve nausea and vomiting. In this study, stimulation reduced episodes of vomiting from 1 per week to 1 per month in approximately 30% of subjects. Temporary stimulation methods have been proposed by some centers to help improve patient selection, and to personalize stimulation parameters and lead placement (Abell et al., 2011; Elfvin et al., 2011; Corvinus et al., 2018).

Gastric stimulation protocols have also been applied to treat obesity by reducing motility, delaying emptying and increasing satiety. Pre-clinical trials showed that stimulation was able to delay gastric emptying, distention, and reduce food intake and eventually led to weight loss (Song et al., 2015). Initial trials in humans showed encouraging results (Favretti et al., 2004; Bohdjalian et al., 2006), but randomized trials have failed to show clinical benefit over a prolonged period (Shikora et al., 2009; Paulus et al., 2020).

Table 2 summarizes types of stimulation parameters that have been applied in both animal and human studies. There is a large variability in the stimulation parameters compared to gastric pacing protocols (see Table 1). The typical range of pulse amplitude is 2–10 mA, but there was high level of variability in pulse width (typical range 0.2–10 ms), frequency (typical range 0.2–50 Hz), and on/off periods (typical range 0.1–5 s on period/3–55 s off period). A high degree of variation in the stimulation parameters is noted in these studies and it is hypothesized that different sets of parameters target different functional responses. Typically, activation of vagal afferents is achieved at a stimulation frequency of 20–30 Hz and frequencies higher than 50 Hz can potentially cause damage to the nerves (Bonaz et al., 2013). On the other hand, lower than 5 Hz frequency is understood to be more effective at targeting the vagal efferents (Bonaz, 2018). Therefore, parameters with excitatory or inhibitory effects need be accordingly chosen for an effective stimulation. For instance, trains of short pulses were reported to be more effective on nausea and vomiting as it was mediated by vagal pathway (Chen et al., 2003). Similarly, parameters that inhibit antral contractions via the sympathetic pathway would be more effective to treat obesity (e.g., amplitude 10 mA, width 0.6 s, 2 s period, 3 s off period, frequency 40 Hz) (Zhu and Chen, 2005). The effect of different stimulation protocols has also been evaluated using transcutaneous auricular vagal nerve stimulation. In an acute setting, auricular stimulation has been shown to normalize gastric activity when stimulating at 10 Hz, whereas slow wave activity was inhibited when stimulation was applied at 80 Hz (Sukasem et al., 2020).

**TABLE 2:** Protocols used for gastric stimulation.

References	Species	Amplitude	Pulse Width	Frequency	On/Off Period
Bilgutay et al., 1963	Human	7–10 mA	10 ms	50 Hz	5 s/55 s
Moran and Nabseth, 1965	Human	10 mA	10 ms	50 Hz	5 s/55 s
Familoni et al., 1997b	Human	2 mA	0.3 ms	0.2 Hz	Not specified
De Luca et al., 2004	Human	10 mA	0.208 ms	40 Hz	2 s/3 s
Abell et al., 2002	Human	5 mA	0.330 ms	14 Hz	∼0.07 s/∼5 s
Angeli et al., 2016	Human	5–19.2 mA	0.33, 0.45 ms	14–130 Hz	0.1–4 s/1–17 s
Ducrotte et al., 2020	Human	5 mA	0.33 ms	14 Hz	0.1 s/5 s
Grundfest-Broniatowski et al., 1990	Dog	2–15 mA	2–50 ms	6, 20 Hz	Not specified
Familoni et al., 1997a	Dog	2 mA	0.3 ms	0.075–0.5 Hz	30 min on
Qin et al., 2007	Rat	6 mA	0.3, 3 ms	14, 40 Hz	0.1, 2 s/3, 5 s
Tang et al., 2006	Rat	6 mA	0.3, 3 ms	20, 40 Hz	0.1, 2 s/3, 4.9 s

### Alternative Approaches

Alternative approaches for pacing and stimulation include combining stimulation and pacing methods, use of multiple stimulation electrodes, synchronizing the applied pulses with the intrinsic activity, and direct stimulation of the vagus nerve.

Gastric pacing is able to entrain gastric slow waves and normalize gastric dysrhythmias and, in some cases, improve gastric emptying (see section “Gastric Pacing”). However, pacing methods do not improve symptoms of nausea and vomiting (Zhang and Chen, 2006). On the other hand, gastric stimulation has been shown to improve symptoms of nausea and vomiting but not to improve gastric emptying (O’Grady et al., 2009; Ducrotte et al., 2020). The ability to improve both emptying rates and treat symptoms is a key therapeutic goal. Therefore, dual pulse approaches that incorporate both pacing and stimulation methods have been proposed (Liu et al., 2004, 2006b). In rats, stimulation with short pulses (e.g., 0.3 ms) and pacing with long pulses (e.g., 550 ms) were reported to improve gastric emptying and alter slow waves patterns (Liu et al., 2004). Similarly, when applied in a canine study, both gastric dysrhythmias and symptoms were normalized by the dual pulse approach (Liu et al., 2006b).

The use of multiple sites for stimulation or pacing has been proposed to influence a larger region of the stomach (Song et al., 2005; Lin et al., 2011). Such approaches have successfully modulated a larger region of the stomach using two pairs for leads utilizing lower levels of total energy compared to a single lead approach. By implanting up to six rings of stimulating electrodes circumferentially around the stomach and stimulating sequentially, it was possible to induce circumferential contraction patterns and increase gastric emptying (Mintchev et al., 2000).

In most studies, pacing and stimulation are performed at a fixed frequency or in an “open-loop” fashion irrespective of the underlying slow wave frequency. In many gastric pacing studies, the intrinsic frequency is determined in a baseline recording period, and then pacing applied at a slightly higher frequency (see Table 1). However, the timing at which these pulses are applied is still irrespective of the underlying activity. Studies have proposed “closed-loop” or synchronized methods which sense the underlying activity and then stimulate at appropriate times (Wang et al., 2020a, b). Experimentally, the synchronized methods were able to either induce or enhance antral contractions and increase rates of gastric emptying (Zhu et al., 2007).

Obesity has also been a target for gastric stimulation and pacing (see section “Obesity”) and some human trials have reported promising results (Cigaina, 2004; Favretti et al., 2004; Yao et al., 2005; Liu et al., 2006a). The most comprehensive study has used vagal nerve stimulation to provide a significant benefit for obesity via the Maestro Rechargeable system or VBLOC (Johannessen et al., 2017). A double-blinded randomized controlled study with 53 subjects receiving the therapy and 31 in a sham group has shown that vagal nerve stimulation resulted in a significantly greater weight loss. At 12 months, the percentage excess weight loss was 33% for those that received the VBLOC therapy compared to 19% in the sham group (Morton et al., 2016). Despite promising outcomes from these stimulation and pacing approaches, further controlled studies are required to establish reliability.

## Mathematical Models

As described in section “Gastric Stimulation and Pacing,” a large variation of stimulation or pacing parameters are used and there is no consensus of parameters that result in the best functional outcomes. Given the range of different gastric electrical stimulation protocols and the inherent dynamic responses to stimulation, a stable platform is required to optimize the protocols in order to minimize the number of animal and human subject trials. An *in silico* Virtual Stomach model is an attractive strategy for systematically evaluating the electrophysiological and functional responses to stimulation protocols (Cheng et al., 2013; Du et al., 2013). Readers are encouraged to follow a previous review of the state-of-art in multi-scale modeling development of gastrointestinal electrophysiology (Corrias et al., 2013; Du et al., 2018). However, there has been relatively limited use of mathematical models to investigate the influence of external stimulation.

One of the earliest modeling studies that investigated the effects of gastric stimulation utilized a conoidal dipole model of whole-organ gastric slow wave activity, and four rings of stimulating electrodes (2 s bipolar trains of 50 Hz, 15 V) were capable of entraining contractions in the distal region of the stomach (Mintchev and Bowes, 1997). While the study assumed a relatively simple geometry of the stomach, the effects of the stimulation protocols were validated in canine subjects. The conoidal model was then refined to incorporate acetylcholine as the neurogenic pathway to drive mechanical response to stimulation (Rashev et al., 2002). The simulation results suggested that rectangular bipolar stimulating trains are potentially more effective in depolarization of smooth muscle cells because of the increased likelihood of matching externally facilitated cell depolarization with invoked release of acetylcholine (Rashev et al., 2002).

A series of automata or rule-based models were also developed to investigate the effects of pacing or slow wave abnormalities in dogs, pigs, and rats (Familoni et al., 2005; Du et al., 2009b; Lammers et al., 2011). One of the key features of the rule-based models was the ability to impose precise definitions of the relative and absolute refractory periods so the expected propagations and their responses to stimulation could be simulated quickly and the system can be adopted to model a closed-loop operation (Wang et al., 2020a). However, a limitation of this approach is the plasticity of the response of the individual automata, which means frequency dependent responses to stimulation, such as frequency restitution cannot be easily modeled (Wang et al., 2017).

Advances in computational power have enabled the development of biophysically based models that are able to more accurately represent the underlying physiology. These models have been used to investigate the slow wave propagation over detailed tissue block representations and the whole stomach (Corrias et al., 2013; Du et al., 2018). A recent study has included anatomically realistic representations of a human stomach and fiber architecture to investigate pacing parameters on slow wave entrainment (Sathar et al., 2015).

Vagal nerve stimulation has been shown to offer therapeutic benefit for modulating gastric function (see section “Electrical Conduction System of the Stomach”) (Payne et al., 2019; Ward et al., 2020). However, mathematical models of the enteric nervous system have largely focused on interaction with the small intestine (Chambers et al., 2014; O’Sullivan-Greene et al., 2018). By modeling vagal afferent signaling of intestinal inflammation, O’Sullivan-Greene et al. (2018) showed that the information delivered to the central nervous system can be predicted and potentially be used in feedback control of stimulators. In order to parameterize and personalize vagal stimulation models various detailed physiological metrics may be required. One study has investigated the influence of neurostimulation strategies for enhancing small intestine motility patterns (Barth et al., 2017) and determined that stimulation at 0.5 Hz was effective in modulating the intrinsic pacemaker activity of ICC, increasing peristalsis, and reducing overall colonic transit time. Translating these methods to the stomach and incorporating it into patient-specific stimulator may allow for improved benefit.

## Future Directions and Conclusion

Significant advances have been made in our understanding of the mechanisms underlying gastric motility since the first electrical recordings reported in the 1920s (Alvarez, 1922c). Significant research has also been devoted to the application of electrical stimuli to modulate gastric function. However, gaps remain in our knowledge and clinical acceptance remains limited (Yin and Chen, 2008; Paskaranandavadivel et al., 2020).

Recently, significant research programs have been initiated by the NIH, DARPA and companies to address these short comings (Reardon, 2014; Waltz, 2016). The SPARC program initiated by the NIH^2^ has a primary goal to transform our understanding of nerve-organ interactions to advance bioelectronic medicine in a number of organ systems. In the following sections, we discuss possible future directions for gastric stimulation and pacing, modeling efforts as well as other applications in the gastrointestinal tract. It is expected that techniques can be readily translated to other sections of the gastrointestinal tract and organ systems (Avci et al., 2020).

### Future Directions for Stimulation and Pacing

Electrical stimulation and pacing techniques have been successfully applied to treat a number of conditions, with cardiac electrophysiology disorders being the most noted. A number of key challenges need to be addressed before electrical stimulation and pacing can be more widely applied clinically to treat gastrointestinal disorders. Most importantly, there have been limited randomized controlled trials that have carefully evaluated the efficacy of various approaches.

As discussed in section “Gastric Stimulation and Pacing,” there remains a wide variety of stimulation and pacing protocols. In addition, there is variability in the electrode types and electrode placements used. Greater certainty in “optimal” pulse parameters and electrode placements would help to standardize methods. However, it is likely that parameters will need to be specifically personalized for individual subjects prior to implantation and improved selection of patients that will response to such treatments will help to improve overall response rates (Abell et al., 2011).

To achieve this, methods to sense and stimulate the stomach which do not require surgical intervention have also been investigated. Such approaches have already been applied to the Enterra Therapy, whereby a temporary stimulation phase can be trialed to tune parameters and evaluate outcomes prior to eventual stimulator implantation (Ayinala et al., 2005; Abell et al., 2011). Studies have also placed electrodes endoscopically to record the slow wave activity from the mucosal surface of the stomach for up to 5 days (Coleski and Hasler, 2004; Paskaranandavadivel et al., 2019). Attempts have also been made to develop miniature, wireless stimulators that can be deployed endoscopically on the mucosal surface, avoiding the need for surgical implantation (Deb et al., 2012; Kim et al., 2020). Trials have proven successful on pigs, and these techniques await clinical translation.

Entrainment mapping techniques described in section “High Resolution Slow Wave Mapping” show great promise for quantifying the efficacy of pacing techniques to alter slow wave patterns. However, to date, they have only been evaluated in short term studies in pigs (O’Grady et al., 2010b). More detailed evaluation in human subjects with functional motility disorders are urgently needed, as well as evaluation in conjunction with gastric function.

### Future Directions for Modeling

Mathematical models provide an ideal technique to test hypotheses and aid in the interpretation of experimental recordings. Modeling techniques have been widely used in the cardiac field (Pullan et al., 2003; Henriquez, 2014). Over the years, detailed mathematical models of cardiac activity that bridges cellular, tissue and organ level activity have been developed (Trew et al., 2006). These models have been instrumental for guiding the development on new drugs and therapies (Brennan et al., 2009; Bear et al., 2018).

Mathematical models of gastric slow wave activity are also maturing rapidly (Cheng and Farrugia, 2013; Cheng et al., 2013; Du et al., 2013). However, the use of mathematical modeling to study other aspects of the stomach, such as neural regulation, biomechanics and luminal flow patterns is relatively new. A number of ICC and smooth muscle cell models have been developed to simulate normal and abnormal function (Lees-Green et al., 2011; Mah et al., 2020). Anatomically realistic models have been developed over which slow wave function can be simulated (Cheng et al., 2007, 2013). More recently, mathematical models have provided key insights into how intrinsic slow wave frequencies, tissue conductivities and external stimuli influence the initiation, maintenance and termination of gastric slow wave re-entry (Gizzi et al., 2010; Du et al., 2014). As discussed in section “Mathematical Models,” further models incorporating enteric nervous system function are required, along with integration with existing models of slow wave activity.

The use of biomechanics to model stomach function is an emerging research area (Brandstaeter et al., 2019). This is in part due to the large deformations that the smooth muscle can undergo and the relatively complex shape and regional variation of the stomach. Most studies on the gastrointestinal tract have focused on modeling the biomechanics of the esophagus and small intestine, possibly due to their simpler uniform tubular structure (Gregersen and Kassab, 1996; Kou et al., 2017). A limited number of studies have investigated the passive and active mechanical properties of the stomach wall (Jia et al., 2015; Aydin et al., 2017; Tomalka et al., 2017). However, further studies are required to investigate the variation in different regions of the stomach and in different species (Bauer et al., 2020). The most comprehensive anatomically realistic stomach model incorporated electro-mechanical coupling, with explicit representation of ICC and smooth muscle cell activity (Klemm et al., 2020). Mechanic-electrical feedback-mechanism was also included that takes into account the mechanical response due to stretch of the stomach wall.

Computational fluid dynamics (CFD) models provide a method to interpret and quantify the consequences of gastric motility patterns. Significant advances have been made since the initial two-dimensional models were developed (Pal et al., 2004, 2007). Subsequently, anatomically realistic three-dimensional models have been introduced and the influence of enhanced contraction patterns in the distal antrum were investigated (Ferrua and Singh, 2010; Berry et al., 2016). Most recent models have incorporated gastric emptying into the models (Harrison et al., 2018; Ishida et al., 2019). Despite significant advances in CFD models, a number of challenges remain (Brandstaeter et al., 2019). All models to date, have utilized idealized and simplified representations of the stomach anatomy and contraction patterns. More advanced models have used experimental slow wave data to derive contraction patterns, although these remain relatively simplified (Berry et al., 2016; Ishida et al., 2019). Most models assume a rigid wall that deforms in a pre-determined manner. In addition, to reduce the complexity of the stimulations, the contents of the stomach assume simple fluids and generally do not attempt to model the breakdown of solid particles. Only a few models have attempted to model gastric emptying, in part because the interaction between the gastric contractions and pylorus function is poorly understood (Imai et al., 2013; Yokrattanasak et al., 2016). Recent MRI studies have shown the ability to accurately capture dynamic data from the stomach (Bharucha et al., 2011; Lu et al., 2017; Menys et al., 2019). This data provides the opportunity to incorporate more realistic boundary conditions for CFD modeling and provide an integrated understanding of the complex interactions between gastric motility patterns, mixing and emptying (Hosseini et al., 2020).

## Conclusion

Since initially being proposed in the 1960s, significant advances have been made towards the use of electrical therapies for modulating gastric function. However, widespread clinical usage remains limited when compared to the large number of therapies approved to treat cardiac electrophysiology disorders. A number of key challenges remain to be addressed. These include the development of methods to reliably quantify functional responses to electrical therapies, and the convergence of the range of pacing and stimulation protocols that are able to sustain long-term responses. The use of mathematical modeling techniques has the potential to help reduce the number of pulse parameters that need to be tested and optimized in animal and clinical trials. The customization of therapies to a specific individual may also help to provide improved long-term outcomes.

## Author Contributions

All authors drafted and edited the manuscript text. LC, NN, and NP prepared the figures and tables.

## Conflict of Interest

LC, PD, and NP hold intellectual property/patent applications in the field of mapping gastrointestinal electrophysiology. The remaining authors declare that the research was conducted in the absence of any commercial or financial relationships that could be construed as a potential conflict of interest. The handling editor declared a past co-authorship with one of the authors PD.

## Footnotes

1 https://repository.niddk.nih.gov/studies/gpcrc/

2 https://sparc.science/
